# Macromolecular structure phasing by neutron anomalous diffraction

**DOI:** 10.1038/srep31487

**Published:** 2016-08-11

**Authors:** Maxime G. Cuypers, Sax A. Mason, Estelle Mossou, Michael Haertlein, V. Trevor Forsyth, Edward P. Mitchell

**Affiliations:** 1 Faculty of Natural Sciences, Keele University, Staffordshire, ST5 5BG, United Kingdom; 2 ILL, 71 avenue des Martyrs, 38000 Grenoble, France; 3 ESRF, 71 avenue des Martyrs, 38000 Grenoble, France

## Abstract

In this report we show for the first time that neutron anomalous dispersion can be used in a practical manner to determine experimental phases of a protein crystal structure, providing a new tool for structural biologists. The approach is demonstrated through the use of a state-of-the-art monochromatic neutron diffractometer at the Institut Laue-Langevin (ILL) in combination with crystals of perdeuterated protein that minimise the level of hydrogen incoherent scattering and enhance the visibility of the anomalous signal. The protein used was rubredoxin in which cadmium replaced the iron at the iron-sulphur site. While this study was carried out using a steady-state neutron beam source, the results will be of major interest for capabilities at existing and emerging spallation neutron sources where time-of-flight instruments provide inherent energy discrimination. In particular this capability may be expected to offer unique opportunities to a rapidly developing structural biology community where there is increasing interest in the identification of protonation states, protein/water interactions and protein-ligand interactions – all of which are of central importance to a wide range of fundamental and applied areas in the biosciences.

The direct visualisation of protonation states and water networks surrounding biological macromolecules has been a longstanding challenge in structural biology^1^. Neutron protein crystallography is a developing technique that is able to yield this information in a way that relates directly to biological structure and function^2^,^3^,^4^. At the ILL, major developments continue for neutron protein crystallography – these are mostly focused on the LADI Laue instrument that covers larger protein systems^5^,^6^. Smaller systems can be studied at high resolution using the monochromatic D19 diffractometer^7^,^8^. Further investments are also being made at current and future neutron sources and high priority is being placed on their exploitation for structural biology. Examples include the High-Flux Reactor (HIFR)^9^ and Spallation Neutron Source (SNS) at Oak Ridge National Laboratory^10^, and at the J-PARC neutron source in Japan^11^. The European Spallation Source (ESS) is currently being built in Sweden and will feature the NMX protein diffractometer as one of its first instruments.

The insights provided are crucial for an understanding of enzymatic catalysis, redox reactions, and protein engineering relevant to areas such as bio-fuel cells, biosensors and biochips, and drug design. Here we show that it is possible to use neutron anomalous scattering to phase protein crystal structures in a practical fashion. This study derives from early pioneering work in which Schoenborn and colleagues measured anomalous differences in myoglobin-Cd crystals^12^, as originally suggested by Ramaseshan^13^,^14^. This was later extended to a two-wavelength approach by Bartunik^15^. Since then, neutron facilities have evolved, becoming more brilliant and using increasingly efficient detector systems; this, with the ready availability of perdeuteration technologies for the preparation of optimised protein samples^16^,^17^, opens the door to practicable phasing of protein crystal structures using neutron anomalous diffraction. Beyond reactor neutron sources, this capability is likely to be also of interest to researchers that are operating and developing instrumentation at spallation neutron sources, which provide high performance time-of-flight technologies and therefore offer precise energy discrimination. This permits an optimised approach for neutron anomalous scattering. However, the application of this technique has not yet been seriously developed as an approach that could become readily available to the structural biology community.

## Results

**Figure 1:**
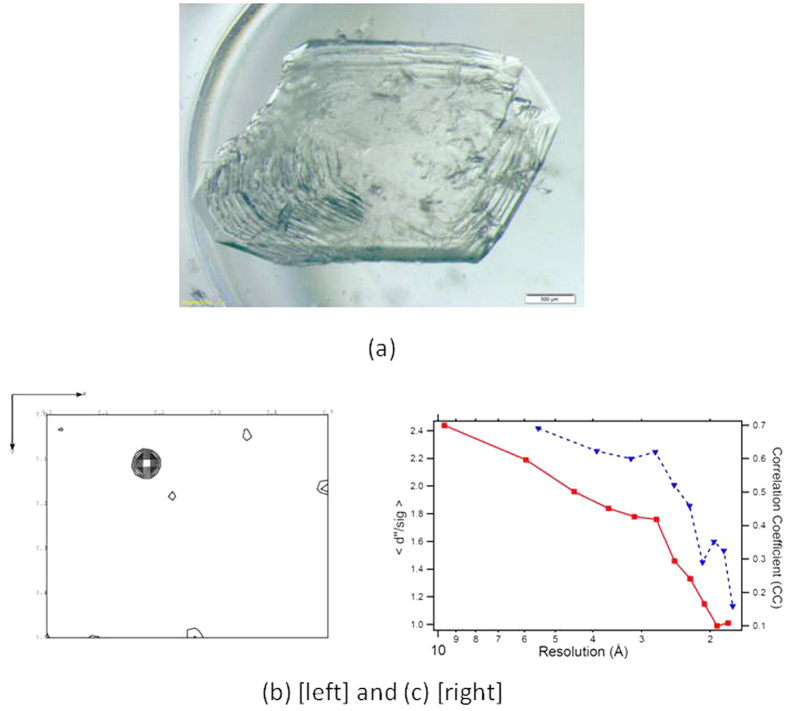
(**a**) The 4.5 mm^3 113^Cd substituted crystal as obtained in the sitting drop container. Scale bar is 100 μm. (**b**) Harker section at z = 0.5 from the neutron Patterson map computed by PATTERSON/CCP4i^32^. Contours levels are shown from 2 to 10 sigma. (**c**) Plot of the anomalous signal to noise ratio (<d”/sig>) obtained from SHELXC (red line) and correlation coefficient as a function of resolution (blue line, from CCP4).

In the current study, perdeuterated *Pyrococcus furiosus* rubredoxin^18^ was produced with the iron atom from the [Fe-4S] cluster replaced by the ^113^Cd isotope. Neutron diffraction data were collected from a perdeuterated crystal (Fig. 1a) using ILL’s high flux thermal diffractometer D19. This instrument has fixed wavelength options of 0.95 Å, 1.17 Å, 1.46 Å and 2.42 Å. The wavelength dependence of the imaginary part of the neutron anomalous dispersion is relatively smooth and is still significant over the hot and thermal neutron range of 0.3 to 3 Å wavelength. For this study a neutron wavelength of 1.17 Å was selected on the basis of a compromise between the proximity to the Cd absorption resonance at 0.68 Å^19^, and the difficulty of resolving Bragg reflections at short wavelength. Diffraction data were collected at room temperature up to 1.75 Å resolution and were corrected for neutron attenuation arising almost solely from the single ^113^Cd atom in the unit cell. The quality of the neutron data is shown by the low value of the precision indicating (multiplicity weighted) R_p.i.m._ value of 14.9% for the highest resolution shell and 4.2% overall (see Table 1).

The data were analysed for the presence of an anomalous signal using SCALA^20^ and SHELXC^21^, which both showed a high anomalous signal-to-noise ratio across most of the resolution range (Fig. 1b). Harker sections calculated from the anomalous Patterson map show a strong 9.0-sigma peak corresponding to the ^113^Cd atom (Fig. 1c). Furthermore, the ratio of R_anom_ values overall and across most of the resolution range to R_p.i.m._ values are greater than 1, indicating an anomalous signal (though care has to be taken in interpretation as R_anom_ does not take into account redundancy)^22^,^23^. The X-ray data can be used as a basis for comparison, as it also contains an anomalous signal. The equivalent Harker section calculated using the X-ray data with the same resolution range as the neutron data (data to 1.75 Å) has a corresponding peak of 7.9-sigma. The comparison against the X-ray data is interesting as both data sets were collected at wavelengths remote from the anomalous edges concerned (1.17 Å experiment wavelength against 0.68 Å for neutrons, and 0.98 Å against 3–3.5 Å for the X-ray LI-LII-LIII anomalous edges). To try to compare the experimental results against the theoretical anomalous signal, the expected anomalous signal as a contribution to |F| was estimated using ΔF/F ≈ √2. (√N_a_.Δb”)/(√N_p_.b_av_), where N_a_ is the number of anomalously scattering atoms, N_p_ is the number of protein atoms, b_av_ is the average neutron scattering length for a protein atom and b” is the imaginary contribution to the scattering length at the wavelength of interest (as adapted from Hendrickson and Teeter^24^). The estimation is 6.7% irrespective of the scattering angle (neutron scattering amplitudes do not fall off appreciably with increasing scattering angle, since the atomic nuclei are effectively point scatterers, if the effects of atomic displacement parameters are not considered). This compares against the measured R_anom_ (which compares reflection pairs for their experimentally measured intensity differences) of 7.5% overall. Care needs to be taken as the estimation is based upon |F| whilst the measurement is based upon I. The equivalent figures for the X-ray data are 1.5% at 10 Å resolution and 2.8% at 1.75 Å resolution and an R_anom_ of 2.8%.

**Figure 2:**
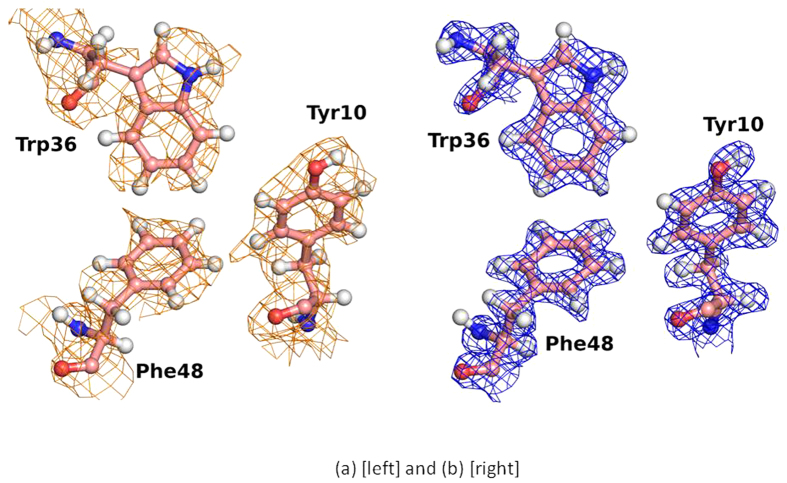
The neutron scattering density maps near the hydrophophobic core of *Pf* rubredoxin (Trp36, Tyr10, Phe48) rendered with PYMOL^43^ are shown. (**a**) The experimental phased map from SHELXE (2.30 Å resolution, contoured at 0.8 sigma in orange). (**b**) The 2Fo-Fc map from the refined final model (1.75 Å resolution, contoured at 1.5 sigma in blue).

SHELXC/D/E^21^ were used to determine the coordinates of the ^113^Cd atom and to phase a neutron Fourier map. This experimental density map is of high quality and shows clear features corresponding to the protein structure (Fig. 2a). Using the phased diffraction data, SHELXE automatically built an initial model of 38 polyalanine residues out of a total of 54. Cycles of manual construction and refinement gave a final model consisting of all 54 residues and 49 D_2_O molecules with R/R_free_ of 23.3/28.6% (see Table 1). The overall correlation coefficient between the original SHELXE experimentally phased map and the final refined model calculated phased map is 60.3% including all data to 2.30 Å resolution.

## Discussion

The use of neutron anomalous diffraction differences was first suggested in the 1960s for crystals containing atoms such as ^113^Cd, ^149^Sm, ^151^Eu and ^157^Gd^10^. At the time, an anomalous neutron data collection experiment required very large crystals and fewer than 100 reflections could be measured per day; hence experiments were very expensive in neutron beam time and far from tenable as a realistic phasing method for proteins. The present demonstration takes advantage of the neutron anomalous scattering properties of ^113^Cd, the availability of modern high-intensity monochromatic neutron beamlines such as D19 at the ILL, and protein perdeuteration to determine the structure of a biological macromolecule using *de novo* phasing in a reasonable timescale. As pointed out by Bacon^19^, isotopic replacement of hydrogen by deuterium atoms in a crystal provides a crucial advantage in all but eliminating hydrogen incoherent scattering and thus enhancing the visibility of the anomalous signal. Advanced facilities that have developed for macromolecular deuteration now permit the production of suitably labelled protein crystals on a routine and reliable basis^16^. It should be noted that this method may provide a useful complement to X-ray anomalous phasing approaches – particularly where structure determination at room or physiological temperature is advantageous^25^. In the current study the anomalous scatterer was introduced in the metal chelating site using chemical reagents to remove the native iron prior to isomorphous replacement with the ^113^Cd isotope. In future studies one can envisage the soaking of anomalous scatterers into macromolecular crystals in a way that is routinely used in X-ray diffraction studies (see for example^26^ and references therein). Anomalous protein phasing work using X-rays can be delicate when carried out at room temperature, and neutron approaches, for which radiation damage effects are essentially negligible, provide a way of tackling these problems. Experiments close to physiological temperatures can highlight structural differences induced by cryo-cooling, and which may be more representative of *in vivo* structures and their resulting functionalities. This is reflected in the recent upsurge of interest in room temperature structures stimulated by FEL studies, including the work of Lui *et al.* who determined a GPCR crystal structure at room temperature^27^. This structure showed distinct thermal motions and residue conformations different to the cryo-cooled structure.

**Figure 3:**
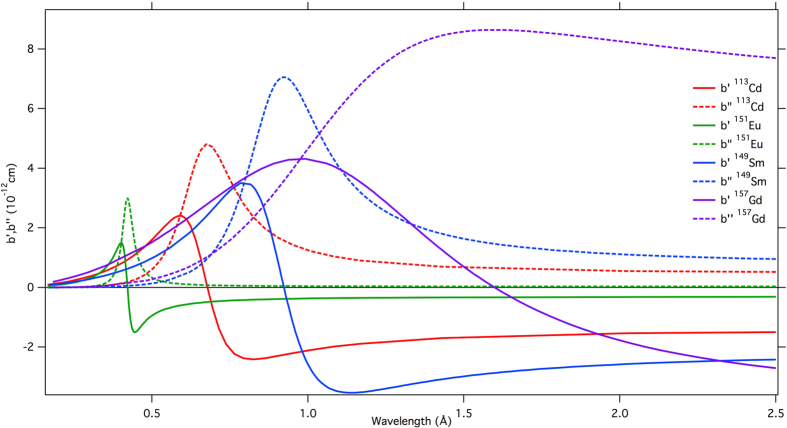
Calculated values of b’ and b” for E = 178 meV neutron resonance of ^113^Cd, ^151^Eu, ^149^Sm and ^157^Gd derived from Breit-Wigner formula with the scattering length 
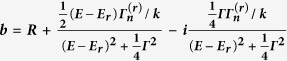
 (E_r_ is the resonance energy, Γ is the total width of the resonance Γ_n_ is the width of resonance for re-emission of the neutron with its original energy, k = 2π/λ is the wavenumber and R the nuclear radius). Resonance parameters E_r_, Γ_n_, Γ were taken from^44^.

The confluence of new spallation neutron sources, high-performance detectors and ready access to perdeutered proteins is paving the way to practical perspectives for neutron phasing methods. The impact of this approach is likely to be greatly enhanced by the fact that time-of-flight measurement at spallation neutron sources will, in a single experiment, provide diffraction data over the whole wavelength range accessible to a given instrument. Plots of the real (b’) and imaginary (b”) components of the neutron scattering length for selected isotopes are provided in Fig. 3, highlighting the availability of an anomalous signal across neutron wavelength ranges. This offers key advantages given that anomalous differences will *de facto* be available over a wide wavelength range encompassing the resonance energies of isotopes that are relevant for the study of biological crystals.

For convenience, standard criteria were used during phasing of the rubredoxin data. We furthermore opted not to carry out solvent flattening approaches so as to minimise assumptions about the structure/solvent in evaluating the phasing power of the system. SHELX was therefore directed to perform zero cycles of solvent flattening and the two resulting neutron scattering density maps (corresponding to the original and inverted hands of the cadmium substructure) were distinguished on the basis of visual inspection, which showed the inverted hand to be the correct one for phasing. This shows that the data and *de novo* phasing were of high quality. In future work on the methodology, it would be desirable to adapt current crystallographic software to better accommodate neutron experiments. This would allow the approach to be fully integrated with standard data analysis packages and be more accessible to the wider scientific community. Furthermore, the increased availability of specialised support laboratories for the production of deuterated protein material and optimised crystal growth would benefit the community at large and further enhance the exploitation of neutron spallation sources in this area.

## Methods

### ^113^Cd-substituted perdeuterated *Pyrococcus furiosus (Pf)* rubredoxin (Ru) preparation

The pET28a plasmid encoding a synthetic gene for *Pf* Ru was obtained from GeneArt (Life Technologies, USA). The perdeuterated iron substituted oxidised form of the protein was produced as described elsewhere^18^,^28^ and validated by mass spectrometry. The apoprotein was obtained using a previously reported procedure^29^, adapted for *Pf* Ru. All buffer constituents except trichloroacetic acid and beta-mercaptoethanol (BME) were filtered through a Chelex resin to remove iron contamination. The Fe-containing protein was denatured on ice with 15% trichloroacetic acid (TCA), 0.5 M beta-mercaptoethanol (BME), with gentle tube inversion for 5 min. The precipitate was recovered by centrifugation at 10000 rpm for 3 min in a table top centrifuge. The white protein precipitate was redissolved for 20 min in 0.5 M Tris base + 60 mM BME. This procedure was repeated twice. To introduce cadmium, an equivalent volume of 25 mM Tris pH7.5 was added to the concentrated protein liquor prior to the addition of 2 molar equivalents of ^113^CdCl_2_ (min. 95% isotope enriched, BuyIsotope, Sweden). The clear protein solution was exchanged using centrifugal concentration devices against D_2_O/Tris 25 mM pD7.5, KCl 150 mM until reaching 99.75% D_2_O theoretical content at ~80 mg/ml of final protein concentration. During this concentration process, excess soluble ^113^Cd was eliminated.

### Crystallogenesis

Crystal growth followed a multi-step process as described below:

1. The ^113^Cd rubredoxin solution (~80 mg/ml, see above) was equilibrated for 48 hours in a sitting drop container against 1:1 volume of 3.8 M NaH_2_PO_4_ : 3.8 M K_2_HPO_4_ in >99.7% D_2_O^28^.
2. Microseeds of Fe-substituted *Pf* D-rubredoxin, obtained according to a method described by Jenney & Adams^28^ and by Cuyper*s et al.*^18^, were added to a pre-equilibrated ^113^Cd rubredoxin solution. Small hybrid crystals of ^113^Cd and Fe *Pf* D-rubredoxin were obtained. These contained the red coloured microseed at the centre and clear ^113^Cd-substituted protein component at the exterior.
3. The ^113^Cd part of one small hybrid crystal (above) was crushed and used to seed growth of purely ^113^Cd substituted crystals by repeating steps 1 and 2 to produce crystals with dimensions in the range of 0.1–0.5 mm.
4. A sitting drop of protein (~120 μl) was pre-equilibrated at room temperature against a well solution of 2 ml of 3.7 M equimolar NaH_2_PO_4_ : K_2_HPO_4_ in a purpose-designed sitting drop container. Numerous small protein crystals (from stage three) were added to the pre-equilibrated sitting drop, and 20–40 μl of pure D_2_O was progressively added until the crystals were completely dissolved. The drop tray was then closed hermetically and allowed to equilibrate for 48 h against the 3.7 M salt solution.
5. A 50 μm large defect-free bipyramidal crystal from the third stage was washed in fresh 3.7 M phosphate buffer in D_2_O and transferred with a nylon loop into the 120 μl pre-equilibrated protein-phosphate buffer container (from stage four). A large crystal of volume 4.5 mm^3^ (Fig. 1a) used for the diffraction study was acquired within 6 days and encapsulated in a 3 mm diameter quartz capillary, sealed with epoxy resin, and mounted on a goniometer head for the neutron diffraction experiment.

### X-ray data collection, processing and model refinement

Verification of the ^113^Cd-substituted protein structure was carried out by X-ray crystal diffraction and X-ray fluorescence prior to the large crystal growth. X-ray data collection was performed at 295 K on ESRF (Grenoble, France) beamline ID23-1^30^ using a very heavily attenuated X-ray beam with a quartz capillary encapsulated crystal. The crystallographic data are summarised in Table 1. Data integration was carried out using XDS^31^. The resulting intensities were merged and scaled with SCALA^32^. Molecular replacement was performed using MOLREP/CCP4^33^ using an X-ray structure of the oxidised form of Fe-*Pf* D-rubredoxin at 295 K (pdb code 4AR5) as the probe. Refinement was performed using data to 1.02 Å resolution with automatic water placement using PHENIX.REFINE^34^ in conjunction with COOT^35^. All atoms, including water oxygens but with the exception of deuterium atoms, were refined with anisotropic atomic displacements. The final model is accessible under PDB code 5AI3.

### Neutron data collection, processing and model refinement

Using instrument D19 at the ILL (Grenoble, France), a complete and highly redundant neutron dataset was recorded using omega step-scans of 0.07° with an exposure time of 50 seconds per image at a neutron wavelength of 1.17 Å for the majority of the images. The merged data contains a 99.9% complete set of reflections with high redundancy to 1.75 Å resolution. The dataset was processed with the ILL program RETREAT^36^ and corrected for effective neutron attenuation using PLATON^37^ with a μ value of 1.54 cm^−1^ at 1.17 Å wavelength (μ = (∑ (coherent absorption cross section) + ∑ (incoherent absorption cross section)) * n atoms/cell volume). The neutron cross section of ^113^Cd used was 19800 (+/−400) barn for 1.17 Å wavelength^38^. The reflection data from RETREAT was converted into mtz format with POINTLESS^39^ using unit cell parameters refined from the X-ray data (a = 34.44 Å; b = 35.14 Å; c = 43.78 Å, α = β = γ = 90°; P2_1_2_1_2_1_). Anomalous pairs were handled separately whilst merging data using SCALA up to a resolution of 1.75 Å. The program MTZ2SCA^40^ was used to convert the neutron anomalous data mtz file into sca file format for the purpose of compatibility with HKL2MAP^41^ and SHELXC/D/E^21^. Data corresponding to a resolution of better than 2.3 Å resolution were excluded from SHELXD for single anomalous dispersion (SAD) calculations. For convenience, SHELXD was directed to search for a single atom of Cd using 10,000 trials and found one position. Density modification was not performed in SHELXE and the resolution was extrapolated to 1.5 Å. The neutron scattering maps calculated using the non-solvent modified experimental phases of the original and inverted hands of the Cd substructure used for the SHELXE run showed clear differences with protein structure features readily identifiable in the map corresponding to the phasing using the inverted hand substructure. This phase set was therefore used thereafter. At the end of the SHELXE run, the correlation coefficient of the best partial structure trace against neutron data was 18.7% with a total of 38 alanine residues built. The output file containing the experimental phases from SHELXE was converted into an mtz file format using F2MTZ (script from T. Grüne). A free Rflag set of reflections of 5% was generated with IMPORT/CCP4. This reflection file was used in ARP/wARP^42^. Neutron data collection took 13 days, with subsequent integration, neutron attenuation correction and phasing achieved using routine crystallography software in ~2 days. The ARP/wARP model was enhanced with cycles of manual building with COOT together with neutron data refinement with a modified scattering table version of PHENIX.REFINE for the ^113^Cd isotope (neutron coherent scattering length density of ^113^Cd is −8.0 fm) with automatic water placement. Model refinement statistics are included in Table 1. The structure has been deposited under PDB accession code 5AI2.

## Additional Information

**Accession codes:** The diffraction data and refined models have been deposited in the Protein Data Bank with accession codes 5AI2 (neutron), 5AI3 (X-ray).

**How to cite this article**: Cuypers, M. G. *et al.* Macromolecular structure phasing by neutron anomalous diffraction. *Sci. Rep.* **6**, 31487; doi: 10.1038/srep31487 (2016).

## Figures and Tables

**Table 1:** Neutron and X-ray data collection characteristics and statistics obtained from SCALA with refined model parameters obtained from PHENIX.REFINE.

Beamline	D19 (ILL)	ID23-1 (ESRF)
**Data collection**		
Type of radiation diffracted	Neutron	Synchrotron X-ray
Crystal volume (mm^3^)	4.5	0.12
Time/frame (s)	50	n/a
Data collection (days)	13	n/a
Temperature (°K)	295	295
Space group	P2_1_ 2_1_ 2_1_	P2_1_ 2_1_ 2_1_
Wavelength (Å)	1.17	0.98
Cell dimensions *a, b, c* (Å)	34.44, 35.14, 43.78	34.44, 35.14, 43.78
Resolution range (Å)	34.44–1.75	43.78–1.02
Rmerge	0.142 (0.457)	0.033 (0.714)
Rp.i.m.	0.042 (0.149)	0.018 (0.341)
Mn *I*/σ(*I*)	12.9 (5.4)	25.4 (3.0)
Wilson B-factor (Å^2^)	n/a	11.24
Mn(I) half-set correlation CC(1/2)	0.996 (0.941)	0.999 (0.704)
Completeness (%)	99.9 (99.9)	91.8 (82.7)
Multiplicity	13.4 (10.6)	6.2 (6.0)
Number observations	74266 (8426)	157832 (19705)
Number unique reflections	5560 (792)	25412 (3282)
Anomalous completeness (%)	100.0 (100.0)	89.8 (80.1)
Anomalous multiplicity	6.6 (5.1)	3.3 (3.1)
Ranom	0.075	0.028
**Refinement**		
PDB code	5AI2	5AI3
Rwork/Rfree (%)	23.3/28.6	10.7/12.6
R.m.s. deviations		
Bonds (Å)	0.003	0.024
Angles (°)	0.613	1.365

Overall values for the selected resolution ranges are presented. Values in parentheses are for the highest resolution shell. Data were collected from one crystal for each structure. Note the X-ray data completeness is 82.7% because of the integration of valid reflections in the corners of the square detector.

Rmerge = (Σ(I − <I>)/Σ(I); where I is the intensity measured for a given reflection, <I> is the average intensity for multiple measurements of this reflection.

Rp.i.m. = (Σ[1/(N − 1)]1/2 Σ|I − <I>|)/Σ(I)^22^

Ranom = Σ |ΔI^±^|/Σ<I>

Rwork = Σ||Fobs| − |Fcalc||/Σ|Fobs|; where Fobs and Fcalc are the observed and calculated structure factor amplitudes, respectively, for 95% of the reflection data used in refinement.

Rfree = Σ||Fobs| − |Fcalc||/Σ|Fobs|; for 5% of the reflection data excluded during the refinement.
